# Appendicular Versus Non-Appendicular Community-Acquired Intra-Abdominal Infections: A Post-Hoc Analysis of the PERICOM Multicenter Study

**DOI:** 10.3390/antibiotics15090910

**Published:** 2026-09-16

**Authors:** Arthur Chouaikhi-Perrois, Nicolas Boulet, Samy Figueiredo, Benjamin Louart, Delphine Garrigue, Guillaume Bouhours, Hervé Dupont, Pierre Bouzat, Jean Bardon, Julien Pottecher, Pierre Michelet, Sébastien Perbet, Katia Aymart, Pascal Incagnoli, Sophie Lloret, Anatole Harrois, Philippe Montravers, Claire Roger

**Affiliations:** 1 UR-UM IMAGINE, Univ Montpellier, Division of Anesthesia and Critical Care, Pain and Emergency Medicine, Nîmes University Hospital, 34090 Montpellier, France; 2 Department of Anaesthesiology and Critical Care, Assistance Publique-Hôpitaux de Paris (AP HP), Université Paris Saclay, 94270 Le Kremlin Bicêtre, France; 3 Surgical Critical Care, Department of Anesthesiology and Critical Care, Centre Hospitalier Universitaire Lille, 59000 Lille, France; 4 Department of Anaesthesiology and Intensive Care, University of Angers, Angers University Hospital, 49933 Angers, France; 5 Department of Anesthesia and Critical Care Medicine, Centre Hospitalier Universitaire Amiens Picardie, SSPC Research Unit, Université de Picardie Jules Verne, 80054 Amiens, France; 6 Pôle Anesthésie-Réanimation, Centre Hospitalo-Universitaire Grenoble-Alpes, Université Grenoble Alpes, 38700 Grenoble, France; 7 Service d’Anesthésie et des Réanimations Chirurgicales, Unité de Réanimation Chirurgicale Neuro-Traumatologique, Assistance Publique—Hôpitaux de Paris, Hôpitaux Universitaires Henri Mondor, 94000 Créteil, France; 8 Pôle d’Anesthésie-Réanimation & Médecine Péri-Opératoire, Service d’Anesthésie Réanimation & Médecine Péri-Opératoire, Hôpital de Hautepierre, Hôpitaux Universitaires de Strasbourg, 67200 Strasbourg, France; 9 EA3072, Fédération de Médecine Translationnelle de Strasbourg (FMTS), Faculté de Médecine, Université de Strasbourg, 67200 Strasbourg, France; 10 Département de Médecine d’Urgence, Hôpital de la Timone—AP-HM, Aix Marseille Université, UMR 1263 C2VN, 13385 Marseille, France; 11 CHU Estaing, University of Auvergne, 63100 Clermont-Ferrand, France; 12 Service d’Accueil des Urgences, Hôpital d’Instruction des Armées Percy, 92140 Clamart, France; 13 Department of Emergency Medicine, Dijon University Hospital, 21000 Dijon, France; 14 Département d’Anesthésie-Réanimation Chirurgicale—DMU PARABOL, Université Paris-Cité, CHU Bichat Claude Bernard, APHP, 75018 Paris, France

**Keywords:** antibiotic therapy, appendicitis, community-acquired, intra-abdominal infection, peritonitis

## Abstract

**Background**: Community-acquired intra-abdominal infections (CA-IAIs) exhibit significant clinical heterogeneity. However, most studies focusing on CA-IAIs do not stratify comparisons by infection site. **Methods**: This post hoc analysis of the prospective multicenter PERICOM cohort (13 French university hospitals, April 2018 to February 2019) included adult patients with CA-IAIs requiring surgical management. We compared clinical characteristics, microbiology, and outcomes between appendicular and non-appendicular sites. Secondary analyses assessed the impact of *Enterococcus* spp., *Pseudomonas aeruginosa*, or fungal documentation, and the theoretical coverage of empirical antibiotic regimens. **Results**: Of the 205 patients, 86 (42%) had appendicular and 119 (58%) had non-appendicular CA-IAIs. Appendicular CA-IAIs involved younger patients with fewer comorbidities and were followed by fewer major postoperative complications (Clavien-Dindo score ≥ 3: 10% versus 43%, *p* < 0.001) and no deaths (0% versus 12%, *p* < 0.001). Among 118 culture-positive patients, appendicular CA-IAIs showed higher rates of non-fermenting Gram-negative bacilli (25% versus 10%, *p* = 0.046) and anaerobes (53% versus 28%, *p* = 0.008) and were more often polymicrobial (76% versus 55%, *p* = 0.020). Conversely, *Staphylococcus* spp. (0% versus 9%, *p* = 0.036) and fungal co-infection (0% versus 12%, *p* = 0.010) were confined to non-appendicular CA-IAIs. Furthermore, *Enterococcus* spp. documentation (24/118, 20%) identified patients with greater admission severity and higher 28-day mortality (33% versus 2%, *p* < 0.001). Amoxicillin-clavulanic acid plus gentamicin covered the isolates of 92% of patients versus 61% for third-generation cephalosporin plus metronidazole (*p* < 0.001), a difference identical in both appendicular and non-appendicular sites. **Conclusions**: Appendicular and non-appendicular CA-IAIs appear to be distinct entities, supporting stratified management. Future guidelines should consider site-specific strategies, therapeutic individualization, and the integration of CA-IAI ecology.

## 1. Introduction

Complicated intra-abdominal infections (IAIs) represent the second leading cause of sepsis in intensive care units (ICUs) and are associated with a mortality rate of 30–35% [1,2,3]. Community-acquired IAIs (CA-IAIs), the most prevalent subtype, primarily result from digestive or biliary tract perforation. However, clinical heterogeneity complicates our understanding of their epidemiology [4]. Specifically, appendicular CA-IAIs differ from non-appendicular locations in that patients are typically younger with fewer comorbidities, exhibit distinct microbiological profiles (e.g., presence of *Pseudomonas aeruginosa*), and have lower morbidity and mortality rates [5,6,7,8]. Despite these distinctions, most studies focusing on CA-IAIs do not stratify epidemiology by infection site [9,10].

Effective IAI management relies on source control and appropriate antimicrobial therapy [11]. Empirical antibiotic therapy is guided by infection severity, comorbidities, immunosuppression, and local epidemiology [4], in accordance with national or international guidelines [12]. Given rising antimicrobial resistance, appropriate empirical antibiotic therapy is critical. Inappropriate initial antimicrobial therapy worsens prognosis, particularly for difficult-to-treat microorganisms such as Gram-negative bacilli (GNB) and fungi [13,14,15,16].

*Enterococcus* spp. are recovered from 5% to 25% of CA-IAIs, almost always within a polymicrobial flora, and their pathogenic role remains contested [17,18,19]. The evidence linking *Enterococcus* to death comes mainly from ICU cohorts and from postoperative peritonitis, where *Enterococcus faecium* has been associated with worse outcomes [18,19]. These series pool community-acquired and healthcare-associated infections, two settings with distinct enterococcal prevalence and resistance ecology. Enterococcal recovery also varies with the anatomical source, and is lowest in appendicular IAIs [20,21]. In complicated intra-abdominal infection, enterococcal recovery predicted treatment failure [20], yet no adequately powered trial has established a benefit of empirical anti-enterococcal coverage in the community setting [19]. Whether community-acquired enterococcal peritonitis defines a distinct population, and whether it carries a distinct prognosis, remains unresolved.

The main objective of this post hoc study of the PERICOM cohort [11] is to describe and compare clinical and microbiological profiles of appendicular versus non-appendicular CA-IAIs.

## 2. Methods

### 2.1. Ethics

The PERICOM study (ID RCB 2017-A03407-46) was approved by the ethics committee “Comité de Protection des Personnes—Est III” (Nancy University Hospital, France). In accordance with French legislation on non-interventional research, the requirement for written informed consent was waived [22]. Patients or their surrogate decision-makers received written information before enrolment and could object to the use of their data at any time. The PERICOM study has been performed in accordance with the Declaration of Helsinki and was registered at ClinicalTrials.gov (NCT03544203).

### 2.2. Study Design and Population

This post hoc analysis uses data from the PERICOM study [11], conducted across 13 French university hospitals, in accordance with the Strengthening the Reporting of Observational Studies in Epidemiology (STROBE) guidelines [23]. This descriptive, prospective, multicenter study included all patients admitted to the emergency department for confirmed CA-IAIs requiring surgical management between April 2018 and February 2019. Primary IAIs, healthcare-associated IAIs, and trauma-related abdominal infections were excluded.

Our clinical analysis included the full cohort. The microbiological analysis included only patients with positive bacterial cultures from peritoneal fluid, bile, abscess drainage, or blood samples. Monomicrobial fungal infections were excluded from the main comparative analyses due to their distinct characteristics. However, they were reintegrated for a specific supplementary analysis comparing all fungal CA-IAIs (monomicrobial and polymicrobial) to non-fungal CA-IAIs.

### 2.3. Data Collection

For each included patient, the following data were collected: demographics (age, sex, weight, height), clinical history (diagnosis, comorbidities, allergies, ICU admission), severity scores at admission (Simplified Acute Physiology Score II (SAPS II) [24] and Sequential Organ Failure Assessment (SOFA) [25]), infection characteristics (infection site, initiated antibiotic therapy, microbiological samples), radiological data, surgical information (approach, duration), and clinical outcomes (Clavien-Dindo classification [26,27], hospital length of stay, 28-day mortality, discharged alive at day 28).

### 2.4. Definitions

Infections were classified as appendicular whenever the appendix was recorded as the infection source, and as non-appendicular otherwise. We used the source of infection recorded prospectively in the PERICOM study and based on perioperative findings [11].

We defined severe IAI as a SOFA score ≥ 2 (binary variable) or the need for ICU admission [12].

The Clavien-Dindo classification was used to grade postoperative complications [26,27]. We defined “major complications” (binary variable) as grade ≥ 3 (requiring surgical, endoscopic, or radiological intervention).

Risk factors for multidrug-resistant (MDR) bacteria comprised antibiotic exposure or hospitalization in the prior 3 months, MDR colonization, and residence in a long-term care facility.

The peritonitis score was determined based on the presence of the following criteria: female sex, upper gastrointestinal tract involvement (gastric, duodenal, or biliary), norepinephrine support, and exposure to antibiotic therapy within three months [28].

### 2.5. Outcomes

The primary objective was to compare clinical characteristics and microbiological profiles of appendicular versus non-appendicular CA-IAIs.

The secondary objectives were to compare clinical characteristics of patients with *Enterococcus*-positive versus other culture-positive CA-IAIs, *Pseudomonas aeruginosa*-positive versus other culture-positive CA-IAIs, fungal-positive (monomicrobial or polymicrobial) versus only bacterial-positive CA-IAIs, and to evaluate the appropriateness of empirical antibiotic therapy and the theoretical in vitro coverage of the four guideline-recommended regimens.

#### 2.5.1. Microbiology Description

Microbiological sampling covered fluids and blood cultures obtained within 24 h of surgical management, and recorded prospectively in the PERICOM study [11].

Microbiological processing was performed locally, in each participating university hospital laboratory, according to routine practice. Species identification and antimicrobial susceptibility testing followed the standard methods in use in each laboratory over the study period, and susceptibility results were interpreted using the European Committee on Antimicrobial Susceptibility Testing (EUCAST) clinical breakpoints [29].

An infection was classified as polymicrobial when more than one bacterial species was isolated. A report of polymorphic anaerobic flora was counted as polymicrobial.

#### 2.5.2. Appropriateness of Empirical Antimicrobial Therapy

Empirical antibiotic therapy was defined as the first regimen administered after patient inclusion. Among patients with bacteriological documentation, we assessed appropriateness for all patients with culture-positive samples. Appropriateness of the regimen administered was adjudicated independently by two investigators from the complete susceptibility report. Therapy was deemed appropriate when every organism considered clinically relevant was susceptible in vitro to at least one administered agent. We also evaluated the theoretical in vitro coverage of the four empirical regimens recommended by the French Society of Anaesthesia and Intensive Care Medicine [12], computed as if every patient had received the regimen, irrespective of the treatment administered.

### 2.6. Statistical Analysis

Continuous variables are reported as medians with interquartile ranges and categorical variables as counts and percentages. Because normality was rejected for all continuous variables (Shapiro–Wilk test, *p* ≤ 0.003 in every subgroup), between-group comparisons used the Wilcoxon–Mann–Whitney rank-sum test, with the Hodges–Lehmann median difference and its 95% confidence interval as the effect measure. Categorical variables were compared with Fisher’s exact test. Differences in proportions are reported with 95% confidence intervals computed by Newcombe’s method. The theoretical coverage of the four guideline-recommended regimens was compared on the same isolates using McNemar’s test for paired proportions. All analyses were performed on complete cases, and the denominator is reported for every variable with missing data. Given the exploratory nature of this post hoc analysis, no adjustment was made for multiple comparisons, and all *p*-values should be interpreted as hypothesis-generating. A two-sided *p*-value below 0.05 was considered statistically significant. Analyses were performed with R version 4.6.0 (R Foundation for Statistical Computing, Vienna, Austria).

## 3. Results

### 3.1. Study Population

**Figure 1:**
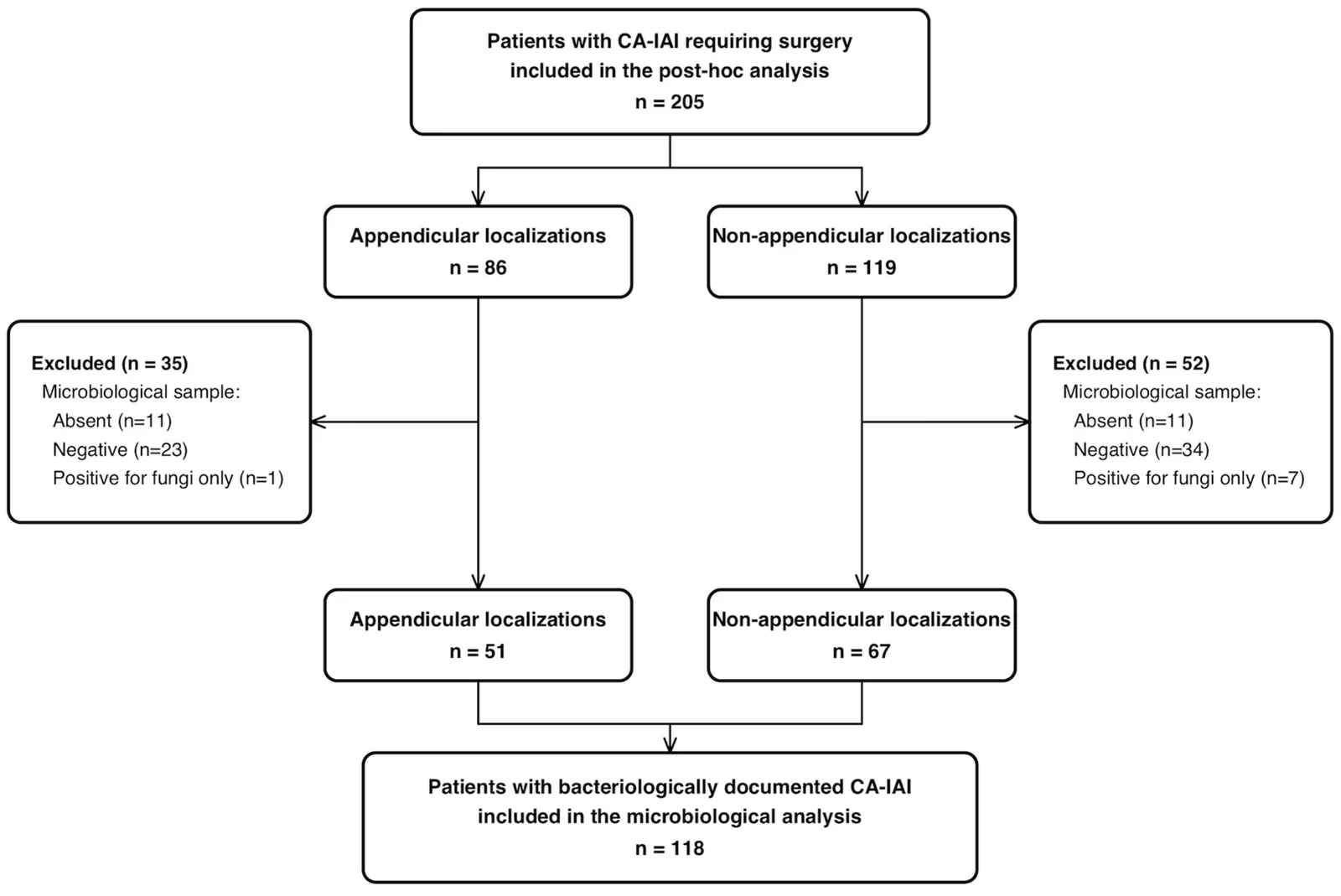
Study flow chart. All 205 patients from the PERICOM cohort were included in the post hoc analysis. A microbiological sample was considered absent when no specimen was obtained from a qualifying site (peritoneal fluid, bile, abscess or intra-abdominal collection, or blood culture). Patients whose only positive culture was fungal were excluded from the main microbiological comparisons and analyzed separately. CA-IAI: community-acquired intra-abdominal infection.

Between April 2018 and February 2019, 205 patients were enrolled in the PERICOM study, all of whom were included in this post-hoc analysis: 86 (42%) with appendicular CA-IAIs and 119 (58%) with non-appendicular CA-IAIs (Figure 1).

### 3.2. Primary Outcome: Appendicular Versus Non-Appendicular CA-IAIs

#### 3.2.1. Clinical Characteristics

Patients with appendicular CA-IAIs were significantly younger, had fewer comorbidities or MDR risk factors, and required ICU admission less frequently (Table 1). Severe CA-IAIs were correspondingly less frequent in this group (6% versus 50%; *p* < 0.001). Imaging and source control were nevertheless obtained later in appendicular CA-IAIs (median imaging delay 4.2 [2.4; 6.6] versus 3.3 [1.1; 5.8] hours, *p* = 0.023; source control delay 11.1 [7.0; 17.6] versus 8.1 [5.1; 14.4] hours, *p* = 0.040). Outcomes differed markedly across the two groups. Patients with appendicular CA-IAIs had shorter hospital lengths of stay (6 [3; 8] versus 9 [5; 20] days, *p* < 0.001), fewer major postoperative complications (Clavien-Dindo score ≥ 3: 10% versus 43%, *p* < 0.001), no death at day 28 (0% versus 12%, *p* < 0.001), and were more frequently discharged alive at day 28 (95% versus 76%, *p* < 0.001).

#### 3.2.2. Microbiological Profiles

**Figure 2:**
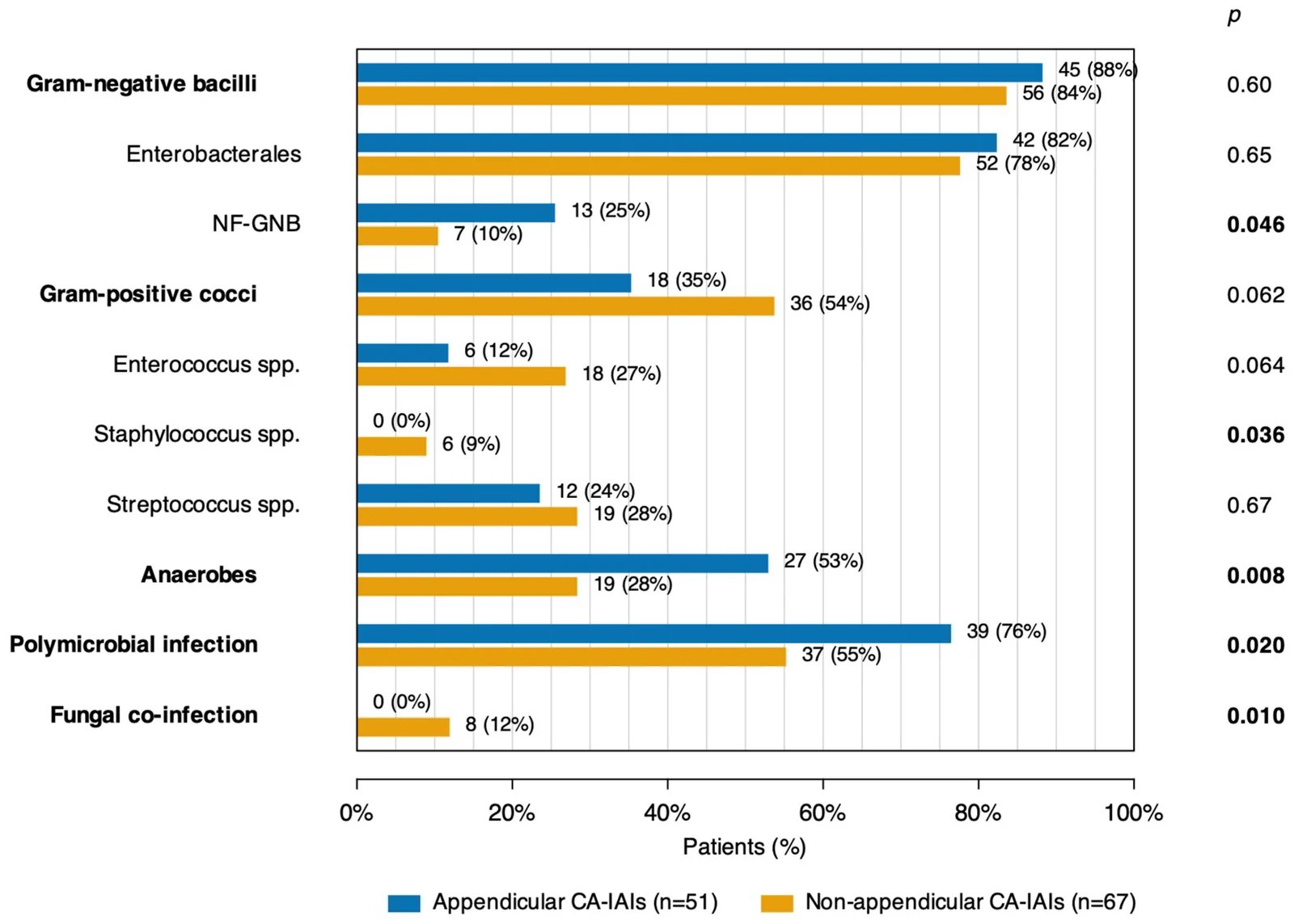
Microbiological profile of appendicular versus non-appendicular CA-IAIs, among the 118 patients with bacteriological documentation. Bars show the number and the proportion of patients in each group (denominators: 51 appendicular and 67 non-appendicular CA-IAIs). Categories are not mutually exclusive, since 76 patients (64%) had a polymicrobial infection. Fungal coinfection refers to the 8 patients with both bacterial and fungal isolates; 8 additional patients with strictly monomicrobial fungal infection were excluded from this comparison. *p*-values are from Fisher’s exact test. Species-level counts are given in Table 2. *p*-values in bold are considered statistically significant (*p* < 0.05). CA-IAI: community-acquired intra-abdominal infection. NF-GNB: non-fermenting Gram-negative bacilli.

Microbiological sampling was performed in 183 (89%) patients, primarily involving peritoneal fluid (n = 159), blood cultures (n = 50), bile fluids (n = 19), and intra-abdominal abscess or collection (n = 9). Patients could contribute more than one sample. Cultures were positive in 126 patients, of whom 16 (13%) had a fungal isolate. Eight strictly monomicrobial fungal infections were excluded from the primary analysis. The remaining 118 patients (58%), including 8 cases of bacterial/fungal coinfection, constitute the microbiological analysis cohort (Table 2, Figure 2).

Microbiological documentation showed higher rates of non-fermenting Gram-negative bacilli (25% versus 10%, *p* = 0.046) and anaerobes (53% versus 28%, *p* = 0.008) in appendicular CA-IAIs, which were also more often polymicrobial (76% versus 55%, *p* = 0.020). Conversely, *Staphylococcus* spp. (0% versus 9%, *p* = 0.036) and fungal coinfection (0% versus 12%, *p* = 0.010) were confined to non-appendicular CA-IAIs, and *Enterococcus* spp. tended to be more frequent in this group (12% versus 27%, *p* = 0.064).

### 3.3. Secondary Outcomes

#### 3.3.1. Appropriateness of Empirical Antimicrobial Therapy

Antibiotic initiation occurred preoperatively in 124 (60%) cases, intraoperatively in 57 (28%), and postoperatively in 24 (12%). Among the 118 patients with positive bacterial cultures, 96 (81%) received appropriate empirical antimicrobial therapy, while 22 (19%) received inappropriate therapy (Table 3). Patients receiving appropriate and inappropriate empirical therapy presented with similar baseline characteristics, comorbidities, and MDR risk factors. Illness severity at admission was also comparable between the two groups, with no significant differences in median SAPS II scores (27 [18; 38] versus 30 [20; 36]; *p* = 0.41), the proportion of patients with a SOFA score ≥ 2 (33% versus 18%; *p* = 0.20), or the need for ICU admission (21% versus 18%; *p* = 1.00). Furthermore, the peritonitis localization (appendicular or not) did not differ significantly between patients who received appropriate treatment and those who received inappropriate treatment (43% versus 45%, respectively; *p* = 0.82). The appropriateness of the initial antibiotic therapy did not significantly impact clinical outcomes, including major postoperative complications (Clavien-Dindo score ≥ 3: 29% versus 32%; *p* = 0.80), hospital length of stay (7 [5; 15] versus 8 [6; 12] days, *p* = 1.00), or 28-day mortality (7% versus 14%; *p* = 0.39). Regarding microbiological profiles, antimicrobial empirical appropriateness varied according to the isolated pathogens. GNB were identified in all cases receiving inappropriate therapy (100%) compared to 82% of appropriate therapy cases (*p* = 0.040). Although non-fermenting GNB (32% versus 14%; *p* = 0.057), anaerobes (50% versus 36%; *p* = 0.33), and Enterococcus spp. (32% versus 18%; *p* = 0.15) were more frequently isolated in patients receiving inappropriate therapy, these differences did not reach statistical significance. No differences were observed for other microbiological categories.

Upon review of the 118 patients with available bacteriological documentation, third-generation cephalosporin plus metronidazole was the most frequently prescribed empirical regimen (42/118, 36%), whereas amoxicillin-clavulanic acid plus gentamicin was used in only 16% of patients (19/118). The empirical combination of amoxicillin-clavulanic acid and gentamicin (108/118; 92%) or piperacillin-tazobactam and gentamicin (109/118; 92%) was more likely to cover the isolated bacteria compared to the combination of third-generation cephalosporin and metronidazole (72/118; 61%) (*p* < 0.001) (Supplementary Figure S1). This difference was identical in both appendicular and non-appendicular CA-IAIs (61% versus 61%, *p* = 1.00). *Pseudomonas aeruginosa* was isolated in 6 of the 22 patients who received inappropriate empirical therapy (27%) versus 10 of the 96 appropriately treated patients (10%) (*p* = 0.076).

#### 3.3.2. Comparison of Enterococcus-Positive Versus Other Culture-Positive CA-IAIs

**Figure 3:**
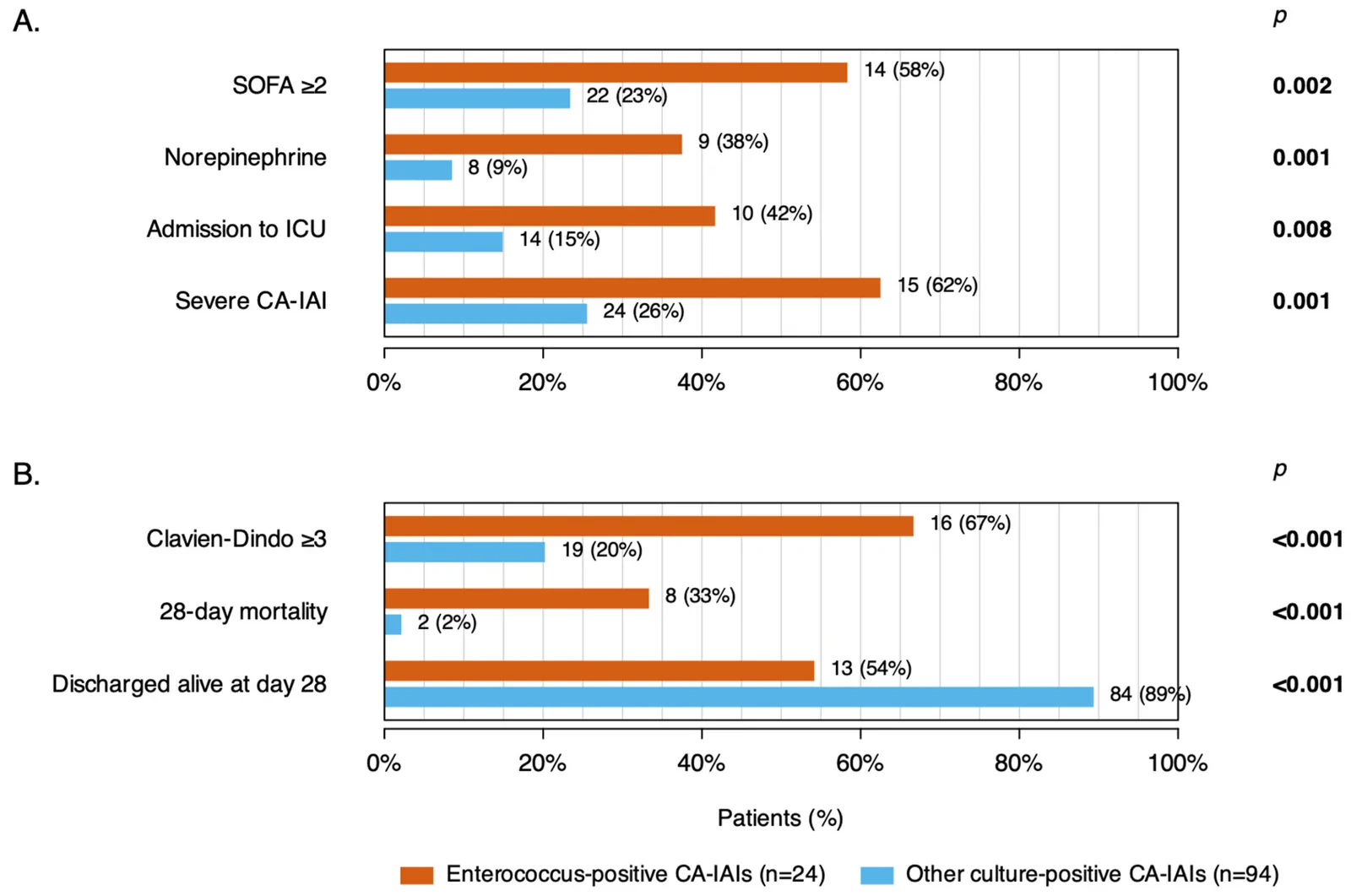
Admission severity (**A**) and outcomes (**B**) of *Enterococcus*-positive versus other culture-positive CA-IAIs, among the 118 patients with bacteriological documentation. Bars show the number and the proportion of patients in each group (denominators: 24 *Enterococcus*-positive and 94 other culture-positive CA-IAIs). *p*-values are from Fisher’s exact test. *p*-values in bold are considered statistically significant (*p* < 0.05). Severe CA-IAI was defined as a SOFA score ≥ 2 or ICU admission. CA-IAI: community-acquired intra-abdominal infection. ICU: intensive care unit. SOFA: Sequential Organ Failure Assessment.

We isolated *Enterococcus* spp. in 24 (20%) of the 118 culture-positive patients (Supplementary Table S1). Compared to patients with other bacterial isolates (n = 94), those with *Enterococcus* spp. presented a higher risk of severe CA-IAI (62% versus 26%; *p* = 0.001), with higher SAPS II scores (38 [27; 44] versus 26 [18; 33]; *p* = 0.003), a greater proportion of patients with a SOFA score ≥ 2 (58% versus 23%; *p* = 0.002), and more frequent ICU admissions (42% versus 15%; *p* = 0.008). Clinically, the *Enterococcus* group experienced significantly worse outcomes, with more major complications (Clavien-Dindo score ≥ 3: 67% versus 20%; *p* < 0.001), higher 28-day mortality (33% versus 2%; *p* < 0.001), and fewer patients discharged alive at day 28 (54% versus 89%; *p* < 0.001) (Figure 3). Notably, the appropriateness of the empirical antibiotic therapy did not differ significantly between groups (71% versus 84%; *p* = 0.15).

#### 3.3.3. Comparison of *Pseudomonas* Aeruginosa-Positive Versus Other Culture-Positive CA-IAIs

*Pseudomonas aeruginosa* was isolated in 16 (14%) of the 118 culture-positive patients (Supplementary Table S2). Its presence was associated with appendicular origin (69% versus 39%; *p* = 0.032) and with polymicrobial infection (94% versus 60%; *p* = 0.010) and tended to be associated with a lower rate of appropriate empirical therapy (62% versus 84%; *p* = 0.076). In contrast, it was associated with no difference in admission severity (SAPS II 24 [19; 37] versus 27 [20; 38], *p* = 0.86; SOFA score ≥ 2: 31% versus 30%, *p* = 1.00; ICU admission 19% versus 21%, *p* = 1.00) and with no difference in any outcome, including major postoperative complications (25% versus 30%; *p* = 0.77), hospital length of stay (7 [6; 14] versus 8 [5; 15] days; *p* = 0.49) and 28-day mortality (6% versus 9%; *p* = 1.00).

#### 3.3.4. Comparison of Fungal-Positive (Monomicrobial or Polymicrobial) Versus Strictly Bacterial Culture-Positive CA-IAIs

Compared to strictly bacterial infections, fungal-positive CA-IAIs occurred in significantly older patients and were characterized by upper gastrointestinal involvement and greater baseline severity, including higher SAPS II scores and a greater need for vasopressors (Supplementary Table S3). These patients underwent laparotomy more frequently (88% versus 41%; *p* < 0.001), experienced more major postoperative complications (Clavien-Dindo score ≥ 3: 56% versus 28%; *p* = 0.041) and stayed in hospital markedly longer (19 [10; 24] versus 7 [5; 14] days; *p* = 0.008), although they had a shorter source control delay (6.0 [3.0; 8.7] versus 10.1 [6.4; 17.8] hours; *p* = 0.006). Finally, 28-day mortality did not significantly differ between groups (19% versus 6%, *p* = 0.12).

## 4. Discussion

This multicenter cohort highlights that appendicular and non-appendicular CA-IAIs represent distinct clinical entities. Patients with appendicular IAIs were significantly younger, had fewer comorbidities, and exhibited better outcomes, supporting the need to stratify these populations in future trials. Conversely, non-appendicular IAIs were associated with higher severity and mortality, driven by older age and comorbidities, which was consistent with previous data [6]. Both imaging and source control occurred later in appendicular CA-IAIs, which is consistent with their lower presenting severity and with emergency department triage priorities.

Microbiologically, our findings align with recent European data, showing a predominance of GNB (86%) [3]. Notably, the appendicular group showed a specific profile with a high prevalence of non-fermenting GNB (25%), consistent with pediatric and adult appendicitis literature [7,8,30]. Although some studies have shown prolonged hospitalization [7,8], the pathogenicity of these microorganisms remains debated in appendicitis. Appendicular CA-IAIs were also more often polymicrobial (76% versus 55%). Together with the excess of anaerobes and of non-fermenting Gram-negative bacilli, this describes a polymicrobial digestive flora released by appendicular perforation, whereas non-appendicular infections more often reflect a single dominant organism from the biliary or upper gastrointestinal tract. This distinction has direct therapeutic implications, since anaerobic and non-fermenting coverage is precisely what separates the guideline-recommended regimens [12].

In our cohort, 19% of patients received inappropriate empirical therapy based on in vitro antimicrobial susceptibility testing, primarily driven by uncovered GNB. Surprisingly, inappropriate therapy was not associated with worse outcomes, contrasting with sepsis literature where delayed effective treatment increases mortality [31,32]. This discrepancy reinforces the concept that high-quality source control remains the primary determinant of survival in CA-IAIs, and provides additional data questioning the pathogenicity of *Pseudomonas aeruginosa* [9,11,33]. Indeed, *Pseudomonas aeruginosa* documentation tended to be more common among patients receiving inappropriate therapy, but without association with any adverse outcome: 28-day mortality, major postoperative complications, and hospital length of stay were all comparable to those of patients without *P. aeruginosa*. This argues against broadening empirical therapy beyond the guideline-recommended regimens to target it in appendicular CA-IAIs.

Nevertheless, our theoretical analysis of regimen coverage challenges the shift toward cephalosporins. The combination of amoxicillin-clavulanic acid with gentamicin provided in vitro coverage in 92% of patients, against 61% for third-generation cephalosporin plus metronidazole. This difference was identical in appendicular and non-appendicular CA-IAIs, indicating that it reflects the intrinsic spectrum of the regimen, which covers neither *Enterococcus* spp. nor non-fermenting Gram-negative bacilli, rather than a site-specific distribution of cases. However, the coverage was calculated in vitro on the isolates actually recovered, as if each patient had received each treatment regimen, and cannot be equated with a rigorous randomized clinical trial. Furthermore, this advantage is largely due to the broadened spectrum associated with the aminoglycoside. These results, therefore, highlight a gap in the spectrum of activity that requires investigation in prospective trials, rather than an established clinical preference.

Another finding of this study is the distinct population identified by the *Enterococcus* spp. documentation. Although routine enterococcal coverage remains debated in CA-IAIs [13,19,30,34], *Enterococcus*-positive patients in our cohort had higher severity scores and worse outcomes, including higher 28-day mortality. These unadjusted subgroup comparisons cannot isolate an independent effect of *Enterococcus*. Enterococcal recovery was itself associated with older age, comorbidity, and non-appendicular sources, and appropriateness of empirical therapy did not differ between groups (71% versus 84%; *p* = 0.15). In line with previous studies, this suggests that *Enterococcus* behaves as a marker of frailty and severity [18,35], identifying patients who warrant closer monitoring, rather than as a pathogen whose systematic empirical coverage is supported by these data [12,36].

Finally, fungal isolation delineated a markedly different population: older, with higher SAPS II scores, more frequent vasopressor support, predominantly upper gastrointestinal involvement (69% versus 22%), and a peritonitis score ≥ 3 in 38% versus 6%. These patients underwent laparotomy more often (88% versus 41%) and stayed in hospital almost three times longer (19 versus 7 days), yet 28-day mortality did not differ significantly (19% versus 6%, *p* = 0.12). Empirical antifungal therapy was given to 62% of them but also to 9% of patients without fungal isolates, indicating that prescription followed clinical suspicion more closely than it followed the peritonitis score.

Finally, fungal isolation delineated a markedly different population: older, with higher SAPS II scores, more frequent vasopressor support, predominantly upper gastrointestinal involvement (69% versus 22%), and a peritonitis score ≥ 3 in 38% versus 6%. These patients underwent laparotomy more often (88% versus 41%) and stayed in hospital almost three times longer (19 versus 7 days), yet 28-day mortality did not differ significantly (19% versus 6%, *p* = 0.12). Empirical antifungal therapy was given to 62% of them but also to 9% of patients without fungal isolates, indicating that prescription followed clinical suspicion more closely than it followed the peritonitis score.

Several limitations temper these findings. This was a post hoc analysis of a prospectively collected cohort; no sample size was computed for the comparisons reported, so our results are hypothesis-generating. The proportion of culture-negative CA-IAIs exceeded that reported in ICU-only cohorts [3,36], likely because less severe cases were included, which further reduced the number of patients available for the microbiological analyses [3,37,38,39]. Subgroup comparisons involved small sample sizes, with results that were likely underpowered. Finally, recruitment was confined to French university hospitals with low baseline resistance rates, which restricts extrapolation to settings with a different ecology.

Nevertheless, this study provides valuable nationwide epidemiological data, emphasizing that appendicular and non-appendicular IAIs require distinct clinical and microbiological considerations in both management and future research.

## 5. Conclusions

This post hoc analysis of the PERICOM study supports the view that appendicular and non-appendicular CA-IAIs are distinct clinical entities with divergent microbiological profiles and outcomes and argues for stratified approaches in future research. To optimize antibiotic stewardship, future guidelines should consider site-specific strategies, therapeutic individualization, and the integration of CA-IAI ecology.

## Figures and Tables

**Table 1:** Characteristics of appendicular and non-appendicular CA-IAI groups.

	Appendicular CA-IAIs (n = 86)	Non-Appendicular CA-IAIs (n = 119)	Difference (95% CI)	*p*-Value
**Demographic characteristics**				
**Age (years)**	46 [29; 66]	65 [50; 76]	−17.0 (−23.0; −10.0)	<0.001
**BMI (kg/m^2^)**	25 [22; 28]	25 [22; 29]	−0.3 (−1.9; +1.0)	0.62
	Missing = 3	Missing = 7		
**Female**	42 (49%)	43 (36%)	+12.7 (−0.9; +25.9)	0.085
Medical history *	29 (34%)	81 (68%)	−34.3 (−46.3; −20.6)	<0.001
**Hypertension**	18 (21%)	47 (39%)		
**Diabetes**	7 (8%)	18 (15%)		
**Chronic kidney disease**	0 (0%)	2 (2%)		
**Ischemic heart disease**	0 (0%)	3 (3%)		
**Chronic heart failure**	2 (2%)	2 (2%)		
**Immunodepression**	2 (2%)	8 (7%)		
**Cancer**	2 (2%)	25 (21%)		
		Missing = 1		
**Obesity (BMI > 30)**	7 (8%)	21 (18%)		
**COPD**	2 (2%)	5 (4%)		
**MDR risk factors**	7 (8%)	26 (22%)	−13.7 (−22.9; −3.6)	0.011
**Admission severity**				
**SAPS II**	20 [12; 27]	31 [20; 40]	−11.0 (−14.0; −7.0)	<0.001
**SOFA ≥ 2**	5 (6%)	52 (44%)	−37.9 (−47.4; −26.8)	<0.001
**Norepinephrine**	1 (1%)	26 (22%)	−20.7 (−29.0; −12.4)	<0.001
**Admission to ICU**	1 (1%)	37 (31%)	−29.9 (−38.8; −20.7)	<0.001
**Severe CA-IAI**	5 (6%)	59 (50%)	−43.8 (−53.2; −32.4)	<0.001
**Management**				
**Imaging delay (hours)**	4.2 [2.4; 6.6]	3.3 [1.1; 5.8]	+1.17 (+0.17; +2.17)	0.023
	Missing = 5	Missing = 6		
**Source control delay (hours)**	11.1 [7.0; 17.6]	8.1 [5.1; 14.4]	+2.13 (+0.10; +4.13)	0.040
**Laparotomy**	9 (10%)	69 (58%)	−47.5 (−57.3; −35.3)	<0.001
**Surgery duration (hours)**	1.2 [0.8; 1.8]	2.0 [1.3; 2.7]	−0.68 (−0.93; −0.43)	<0.001
	Missing = 1	Missing = 3		
**Generalized peritonitis**	23 (27%)	54 (46%)	−19.4 (−31.6; −6.0)	0.005
		Missing = 2		
**Outcomes**				
**Clavien-Dindo score ≥ 3**	9 (10%)	51 (43%)	−32.4 (−42.6; −20.5)	<0.001
**Hospital length of stay (days)**	6 [3; 8]	9 [5; 20]	−4.0 (−6.0; −2.0)	<0.001
**28-day mortality**	0 (0%)	14 (12%)	−11.8 (−18.8; −5.5)	<0.001
**Discharged alive at day 28**	82 (95%)	91 (76%)	+18.9 (+9.4; +27.7)	<0.001

* Medical history was defined as at least one of the following: hypertension, diabetes, chronic kidney disease, ischemic heart disease, chronic heart failure, immunodepression, cancer, obesity (BMI > 30 kg/m^2^), chronic obstructive pulmonary disease. Quantitative values are expressed as median and interquartile range (25th and 75th percentile). Qualitative values are expressed as a percentage. “Laparotomy” includes “conversion to laparotomy”. BMI: body mass index. CA-IAI: community-acquired intra-abdominal infection. ICU: intensive care unit. MDR: multidrug-resistant bacteria. SAPS II: Simplified Acute Physiology Score II. SOFA: Sequential Organ Failure Assessment.

**Table 2:** Microbiological documentation of appendicular and non-appendicular CA-IAI groups, among patients with bacteriological documentation (n = 118).

	Total (n = 118)	Appendicular CA-IAIs (n = 51)	Non-Appendicular CA-IAIs (n = 67)	*p*-Value
**Gram-Negative Bacilli**	101 (86%)	45 (88%)	56 (84%)	0.60
**Enterobacterales**	94 (80%)	42 (82%)	52 (78%)	0.65
*E. coli*	73 (62%)	40 (78%)	33 (49%)	
*Proteus* spp.	6 (5%)	2 (4%)	4 (6%)	
*Klebsiella* spp.	22 (19%)	7 (14%)	15 (22%)	
*E. cloacae*	7 (6%)	0 (0%)	7 (10%)	
*H. alvei*	3 (3%)	0 (0%)	3 (4%)	
*Citrobacter* spp.	3 (3%)	1 (2%)	2 (3%)	
Miscellaneous	6 (5%)	3 (6%)	3 (4%)	
**NF-GNB**	20 (17%)	13 (25%)	7 (10%)	0.046
*P. aeruginosa*	16 (14%)	11 (22%)	5 (7%)	
Miscellaneous	4 (3%)	2 (4%)	2 (3%)	
**Gram-Positive Cocci**	54 (46%)	18 (35%)	36 (54%)	0.062
***Enterococcus* spp.**	24 (20%)	6 (12%)	18 (27%)	0.064
*E. faecalis*	11 (9%)	0 (0%)	11 (16%)	
*E. faecium*	7 (6%)	3 (6%)	4 (6%)	
Miscellaneous	6 (5%)	3 (6%)	3 (4%)	
***Staphylococcus* spp.**	6 (5%)	0 (0%)	6 (9%)	0.036
*S. aureus*	5 (4%)	0 (0%)	5 (7%)	
*Coagulase-negative staphylococcus*	1 (1%)	0 (0%)	1 (1%)	
***Streptococcus* spp.**	31 (26%)	12 (24%)	19 (28%)	0.67
**Anaerobes**	46 (39%)	27 (53%)	19 (28%)	0.008
*Bacteroides* spp.	30 (25%)	19 (37%)	11 (16%)	
*Clostridium* spp.	5 (4%)	1 (2%)	4 (6%)	
**Polymorphic flora**	7 (6%)	4 (8%)	3 (4%)	
Miscellaneous	7 (6%)	2 (4%)	5 (7%)	
Polymicrobial bacterial infection *	76 (64%)	39 (76%)	37 (55%)	0.020
**Fungal coinfection °**	8 (7%)	0 (0%)	8 (12%)	0.010

* An infection was classified as polymicrobial when more than one bacterial species was isolated. A report of polymorphic anaerobic flora was counted as polymicrobial. ° The 8 patients in this row had both bacterial and fungal infections. An additional 8 patients had strictly monomicrobial fungal infections and were excluded from this table and from the main comparative analyses. Percentages are calculated within each column (n = 118 for the Total column, n = 51 and n = 67 for the appendicular and non-appendicular columns, respectively). Due to polymicrobial infections, cumulative percentages of individual species within a taxonomic category may exceed 100%. CA-IAI: community-acquired intra-abdominal infection. NF-GNB: non-fermenting Gram-negative bacilli.

**Table 3:** Comparison of patients having received appropriate versus inappropriate empirical antibiotic therapy, according to antimicrobial susceptibility testing of positive bacterial cultures, among patients with bacteriological documentation (n = 118).

	Appropriate Antibiotic Therapy (n = 96)	Inappropriate Antibiotic Therapy (n = 22)	*p*-Value
**Demographic characteristics**			
**Age (years)**	64 [43; 72]	64 [57; 71]	0.91
**BMI (kg/m^2^)**	24 [22; 28] Missing = 3	27 [24; 29] Missing = 1	0.16
**Female**	38 (40%)	8 (36%)	0.81
Medical history *	56 (58%)	15 (68%)	0.47
**MDR risk factors**	13 (14%)	5 (23%)	0.32
**Admission severity**			
**SAPS II**	27 [18; 38]	30 [20; 36]	0.41
**SOFA ≥ 2**	32 (33%)	4 (18%)	0.20
**Admission to ICU**	20 (21%)	4 (18%)	1.00
**Severe CA-IAI**	35 (36%)	4 (18%)	0.13
**Peritonitis localization**			0.82
**Appendicular localization**	41 (43%)	10 (45%)	
**Non-appendicular localization**	55 (57%)	12 (55%)	
**Outcomes**			
**Clavien-Dindo score ≥ 3**	28 (29%)	7 (32%)	0.80
**Hospital length of stay (days)**	7 [5; 15]	8 [6; 12]	1.00
**28-day mortality**	7 (7%)	3 (14%)	0.39
**Discharged alive at day 28**	80 (83%)	17 (77%)	0.54
**Microbiological documentation**			
**Gram-negative bacilli**	79 (82%)	22 (100%)	0.040
**Enterobacterales**	74 (77%)	20 (91%)	0.24
**NF-GNB**	13 (14%)	7 (32%)	0.057
***P. aeruginosa***	10 (10%)	6 (27%)	0.076
**Gram-positive cocci**	44 (46%)	10 (45%)	1.00
***Enterococcus* spp.**	17 (18%)	7 (32%)	0.15
***Staphylococcus* spp.**	5 (5%)	1 (5%)	1.00
***Streptococcus* spp.**	28 (29%)	3 (14%)	0.18
**Anaerobes**	35 (36%)	11 (50%)	0.33

Qualitative variables are expressed as frequencies (%). Quantitative variables are expressed as median (25th and 75th percentile). * Medical history was defined as at least one of the following: hypertension, diabetes, chronic kidney disease, ischemic heart disease, chronic heart failure, immunodepression, cancer, obesity (BMI > 30 kg/m^2^), chronic obstructive pulmonary disease. BMI: body mass index. CA-IAI: community-acquired intra-abdominal infection. ICU: intensive care unit. MDR: multidrug-resistant bacteria. NF-GNB: non-fermenting Gram-negative bacilli. SAPS II: Simplified Acute Physiology Score II. SOFA: Sequential Organ Failure Assessment.

## Data Availability

The raw data supporting the conclusions of this article will be made available by the authors on request.

## Supplementary Materials

The following supporting information can be downloaded at: https://www.mdpi.com/article/10.3390/antibiotics15090910/s1. Reference [12] is cited in the supplementary materials.

## Abbreviations

CA-IAI	community-acquired intra-abdominal infection
EUCAST	European Committee on Antimicrobial Susceptibility Testing
GNB	Gram-negative bacilli
IAI	intra-abdominal infections
ICU	intensive care unit
MDR	multidrug-resistant
NF-GNB	non-fermenting Gram-negative bacilli
SAPS II	Simplified Acute Physiology Score II
SOFA	Sequential Organ Failure Assessment
STROBE	Strengthening the Reporting of Observational Studies in Epidemiology
