# Supplementary material for: Appendicular Versus Non-Appendicular Community-Acquired Intra-Abdominal Infections: A Post-Hoc Analysis of the PERICOM Multicenter Study
Source: Antibiotics (Basel). 2026 Sep 16;15(9):910. doi: 10.3390/antibiotics15090910 (PMC13603562; doi:10.3390/antibiotics15090910)
Supplement: Supplementary file 1 [file antibiotics-15-00910-s001.zip › antibiotics-4569313-supplementary.pdf]

SUPPLEMENTARY MATERIAL

SUPPLEMENTARY FIGURE S1. Theoretical in vitro coverage of the four empirical antimicrobial regimens recommended by the French Society of Anaesthesia and Intensive Care Medicine [1], among the 118 patients with bacteriological documentation. (A) Whole microbiological cohort. (B) Stratified by peritonitis subtype.

SUPPLEMENTARY TABLE S1. Comparison of *Enterococcus*-positive versus other culture-positive CA-IAIs, among patients with bacteriological documentation (n=118).

SUPPLEMENTARY TABLE S2. Comparison of *Pseudomonas aeruginosa*-positive versus other culture-positive CA-IAIs, among patients with bacteriological documentation (n=118).

SUPPLEMENTARY TABLE S3. Comparison of fungal-positive (with or without bacterial co-infection) versus only bacterial-positive CA-IAIs (n=126).

Supplementary Figure S1. Theoretical *in vitro* coverage of the four empirical antimicrobial regimens recommended by the French Society of Anaesthesia and Intensive Care Medicine [1], among the 118 patients with bacteriological documentation. (A) Whole microbiological cohort. (B) Stratified by peritonitis subtype.

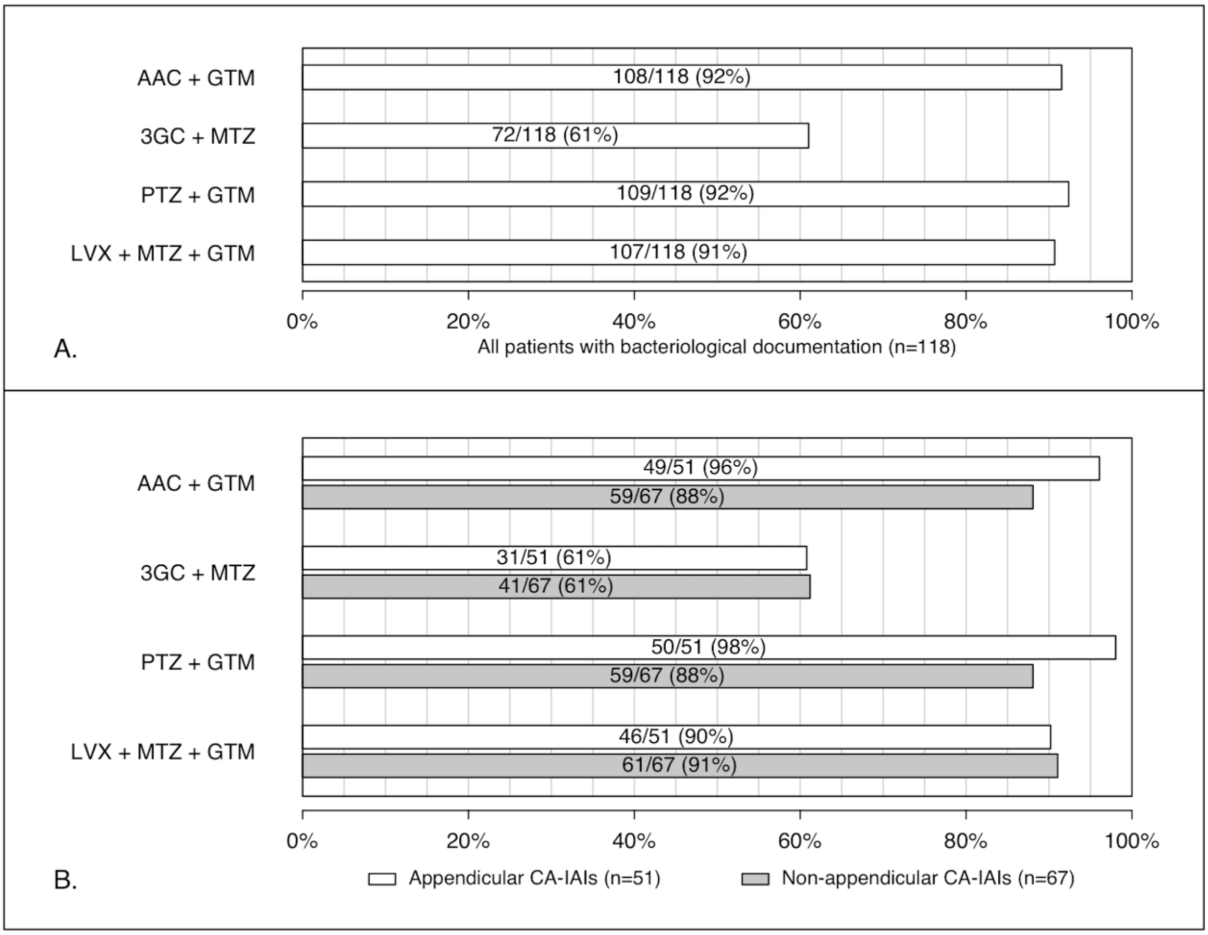

3GC: third generation cephalosporins. AAC: amoxicillin-clavulanic acid. CA-IAI: community-acquired intra-abdominal infections. GTM: gentamicin. LVX: levofloxacin. MTZ: metronidazole. PTZ: piperacillin-tazobactam.

Bars show the number of covered patients over the number of patients in the corresponding group, with the percentage in parentheses.

Theoretical antimicrobial coverage was compared taking AAC + GTM as the reference: 3GC + MTZ, p<0.001; PTZ + GTM, p=1.00; LVX + MTZ + GTM, p=1.00. Appendicular and non-appendicular CA-IAIs were also compared: p=0.18, p=1.00, p=0.076 and p=1.00, respectively.

1. *Montravers P, Dupont H, Leone M, Constantin JM, Mertes PM, Laterre PF, et al. Guidelines for management of intra-abdominal infections. Anaesth Crit Care Pain Med. 2015 Apr;34(2):117–30. doi:10.1016/j.accpm.2015.03.005*

Supplementary Table S1. Comparison of *Enterococcus*-positive versus other culture-positive CA-IAIs, among patients with bacteriological documentation (n=118).

	Enterococcus-positive CA-IAIs (n=24)	Other bacterial culture-positive CA-IAIs (n=94)	p-value
**Demographic characteristics**			
Age (years)	70 [58; 75]	63 [43; 72]	0.13
BMI (kg/m²)	26 [20; 30]	25 [22; 28]<br>Missing=4	0.88
Female	8 (33%)	38 (40%)	0.64
**Medical history***	18 (75%)	53 (56%)	0.11
**MDR risk factors**	7 (29%)	11 (12%)	0.053
**Admission severity**			
SAPS II	38 [27; 44]	26 [18; 33]	0.003
SOFA ≥2	14 (58%)	22 (23%)	0.002
Norepinephrine	9 (38%)	8 (9%)	0.001
Admission to ICU	10 (42%)	14 (15%)	0.008
Severe CA-IAI	15 (62%)	24 (26%)	0.001
**Management**			
Source control delay (hours)	7.7 [5.6; 11.8]	10.2 [6.4; 19.5]	0.21
Laparotomy	15 (62%)	38 (40%)	0.067
Surgery duration (hours)	2.0 [1.0; 2.7]	1.7 [1.1; 2.4]<br>Missing=1	0.70
Generalized peritonitis	12 (50%)	37 (40%)<br>Missing=1	0.49
**Peritonitis localization**			0.064
Appendicular localization	6 (25%)	45 (48%)	
Non-appendicular localization	18 (75%)	49 (52%)	
**Outcomes**			
Clavien-Dindo score ≥3	16 (67%)	19 (20%)	<0.001
Hospital length of stay (days)	8 [6; 16]	7 [5; 13]	0.35
28-day mortality	8 (33%)	2 (2%)	<0.001
Discharged alive at day 28	13 (54%)	84 (89%)	<0.001
**Microbiological documentation**			
Fungal documentation	3 (12%)	5 (5%)	0.20
Appropriate empirical antimicrobial therapy°	17 (71%)	79 (84%)	0.15

Quantitative values are expressed as median and interquartile range (25th and 75th percentile). Qualitative values are expressed as numbers and percentages.

** Medical history was defined as at least one of the following: hypertension, diabetes, chronic kidney disease, ischemic heart disease, chronic heart failure, immunodepression, cancer, obesity (BMI >30 kg/m^2), chronic obstructive pulmonary disease.*

° The data regarding the appropriateness of empirical antimicrobial therapy are expressed relative to the number of patients with bacterial documentation in each group.

BMI: body mass index. CA-IAI: community-acquired intra-abdominal infection. ICU: intensive care unit. MDR: multidrug-resistant bacteria. SAPS II: Simplified Acute Physiology Score II. SOFA: Sequential Organ Failure Assessment.

Supplementary Table S2. Comparison of *Pseudomonas aeruginosa*-positive versus other culture-positive CA-IAIs, among patients with bacteriological documentation (n=118).

	***P. aeruginosa*-positive CA-IAIs (n=16)**	**Other culture-positive CA-IAIs (n=102)**	**p-value**
**Demographic characteristics**			
Age (years)	59 [42; 72]	64 [45; 72]	0.88
BMI (kg/m²)	24 [22; 27]<br>Missing=1	25 [22; 29]<br>Missing=3	0.59
Female	6 (38%)	40 (39%)	1.00
**Medical history***	10 (62%)	61 (60%)	1.00
**MDR risk factors**	4 (25%)	14 (14%)	0.26
**Admission severity**			
SAPS II	24 [19; 37]	27 [20; 38]	0.86
SOFA ≥2	5 (31%)	31 (30%)	1.00
Norepinephrine	4 (25%)	13 (13%)	0.24
Admission to ICU	3 (19%)	21 (21%)	1.00
Severe CA-IAI	5 (31%)	34 (33%)	1.00
**Management**			
Source control delay (hours)	7.6 [6.0; 12.5]	10.2 [6.2; 19.5]	0.23
Laparotomy	10 (62%)	43 (42%)	0.18
Surgery duration (hours)	2.1 [1.2; 2.8]	1.7 [1.1; 2.4]<br>Missing=1	0.35
Generalized peritonitis	6 (38%)	43 (43%)<br>Missing=1	0.79
**Peritonitis localization**			0.032
Appendicular localization	11 (69%)	40 (39%)	
Non-appendicular localization	5 (31%)	62 (61%)	
**Outcomes**			
Clavien-Dindo score ≥3	4 (25%)	31 (30%)	0.77
Hospital length of stay (days)	7 [6; 14]	8 [5; 15]	0.49
28-day mortality	1 (6%)	9 (9%)	1.00
Discharged alive at day 28	12 (75%)	85 (83%)	0.48
**Microbiological documentation**			
Polymicrobial bacterial infection	15 (94%)	61 (60%)	0.010
Fungal documentation	0 (0%)	8 (8%)	0.60
Appropriate empirical antimicrobial therapy°	10 (62%)	86 (84%)	0.076

Quantitative values are expressed as median and interquartile range (25th and 75th percentile). Qualitative values are expressed as numbers and percentages. Continuous variables were compared with the Wilcoxon rank-sum test and categorical variables with Fisher's exact test.

** Medical history was defined as at least one of the following: hypertension, diabetes, chronic kidney disease, ischemic heart disease, chronic heart failure, immunodepression, cancer, obesity (BMI >30 kg/m^2), chronic obstructive pulmonary disease.*
° The data regarding the appropriateness of empirical antimicrobial therapy are expressed relative to the number of patients with bacterial documentation in each group.
BMI: body mass index. CA-IAI: community-acquired intra-abdominal infection. ICU: intensive care unit. MDR: multidrug-resistant bacteria. SAPS II: Simplified Acute Physiology Score II. SOFA: Sequential Organ Failure Assessment.

Supplementary Table S3. Comparison of fungal-positive (with or without bacterial co-infection) versus only bacterial-positive CA-IAIs (n=126).

	Fungal-positive CA-IAIs (n=16)	Bacterial-positive CA-IAIs (n=110)	p-value
**Demographic characteristics**			
Age (years)	74 [61; 85]	63 [43; 72]	0.009
BMI (kg/m^2)	23 [20; 26]	25 [22; 29]<br>Missing=4	0.097
Female	7 (44%)	43 (39%)	0.79
**Medical history***	12 (75%)	64 (58%)	0.28
**MDR risk factors**	5 (31%)	17 (15%)	0.15
**Admission severity**			
SAPS II	42 [36; 54]	26 [18; 34]	<0.001
SOFA ≥2	12 (75%)	30 (27%)	<0.001
Norepinephrine	8 (50%)	14 (13%)	0.001
Admission to ICU	6 (38%)	21 (19%)	0.11
Peritonitis score ≥3°	6 (38%)	7 (6%)	0.002
**Management**			
Source control delay (hours)	6.0 [3.0; 8.7]	10.1 [6.4; 17.8]	0.006
Laparotomy	14 (88%)	45 (41%)	<0.001
Generalized peritonitis	11 (73%)<br>Missing=1	45 (41%)	0.026
Upper gastrointestinal tract involvement	11 (69%)	24 (22%)	<0.001
Empirical antifungal treatment	10 (62%)	10 (9%)	<0.001
**Peritonitis localization**			0.002
Appendicular localization	1 (6%)	51 (46%)	
Non-appendicular localization	15 (94%)	59 (54%)	
**Outcomes**			
Clavien-Dindo score ≥3	9 (56%)	31 (28%)	0.041
Hospital length of stay (days)	19 [10; 24]	7 [5; 14]	0.008
28-day mortality	3 (19%)	7 (6%)	0.12
Discharged alive at day 28	10 (62%)	93 (85%)	0.075

Quantitative values are expressed as median and interquartile range (25th and 75th percentile), while qualitative values are expressed as counts (percentage).
BMI: body mass index. CA-IAI: community-acquired intra-abdominal infection. ICU: intensive care unit. MDR: multidrug-resistant bacteria. SAPS II: Simplified Acute Physiology Score II. SOFA: Sequential Organ Failure Assessment.

** Medical history was defined as at least one of the following: hypertension, diabetes, chronic kidney disease, ischemic heart disease, chronic heart failure, immunodepression, cancer, obesity (BMI >30 kg/m^2), chronic obstructive pulmonary disease.*

° The peritonitis score was determined based on the presence of the following criteria: female sex, upper gastrointestinal tract involvement (gastric, duodenal, or biliary), norepinephrine support, and exposure to antibiotic therapy within three months [Dupont H., et al. Critical Care Medicine 2003]. Prescription of antifungal treatment was considered adherent to guidelines if a patient received antifungal treatment with a peritonitis score of 3 or more.
